# The nuclear import of the transcription factor MyoD is reduced in mesenchymal stem cells grown in a 3D micro-engineered niche

**DOI:** 10.1038/s41598-021-81920-2

**Published:** 2021-02-04

**Authors:** Emanuela Jacchetti, Ramin Nasehi, Lucia Boeri, Valentina Parodi, Alessandro Negro, Diego Albani, Roberto Osellame, Giulio Cerullo, Jose Felix Rodriguez Matas, Manuela Teresa Raimondi

**Affiliations:** 1 Department of Chemistry, Materials and Chemical Engineering “Giulio Natta”, Politecnico di Milano, Milan, Italy; 2 Department of Biomedical Sciences, University of Padua, Padua, Italy; 3 Department of Neuroscience, Istituto di Ricerche Farmacologiche Mario Negri IRCCS, Milan, Italy; 4 Institute for Photonics and Nanotechnologies – CNR, Milan, Italy; 5 Physics Department, Politecnico Di Milano, Milan, Italy

**Keywords:** Biophysics, Computational biophysics, Permeation and transport, Stem cells, Mesenchymal stem cells

## Abstract

Smart biomaterials are increasingly being used to control stem cell fate in vitro by the recapitulation of the native niche microenvironment. By integrating experimental measurements with numerical models, we show that in mesenchymal stem cells grown inside a 3D synthetic niche both nuclear transport of a myogenic factor and the passive nuclear diffusion of a smaller inert protein are reduced. Our results also suggest that cell morphology modulates nuclear proteins import through a partition of the nuclear envelope surface, which is a thin but extremely permeable annular portion in cells cultured on 2D substrates. Therefore, our results support the hypothesis that in stem cell differentiation, the nuclear import of gene-regulating transcription factors is controlled by a strain-dependent nuclear envelope permeability, probably related to the reorganization of stretch-activated nuclear pore complexes.

## Introduction

In the last 2 decades, stem cells have generated a considerable interest in biomedicine with many potential applications such as regenerative and personalized medicine. Mesenchymal stem cells (MSCs) are the most commonly studied due to their ready isolation for autologous use, extensive in vitro expansion capability and the ability to be differentiated into a wide number of tissue-specific lineages^1^.

However, efficient culture systems that allow the large-scale expansion of MSCs while maintaining control of their function are still lacking. Today, much is known about the effect of chemical signals on the activation of cellular biochemical processes. Several studies have shown that also physical signals from the cellular environment, such as substrate stiffness^2^, topography^3^ and dynamic mechanical stimuli^4^, can influence gene regulation and, therefore, cell fate decisions^2–5^. In physiological environments, cells transmit forces from the focal adhesions at their periphery to the nucleus via the actin cytoskeleton, where the nucleus acts as a mechanosensor affecting chromatin organization and gene expression^6^.

While the effect of cytoskeletal deformation on cell fate has been extensively characterised, only recent research has focused on the effects of cell nuclear deformation^7^. For example, it is now accepted that there are mechanosensitive ion channels on the nuclear membrane, which, coupled to the mechanosensitive cytoskeleton, promote ion influx and associated gene transcription^8^. Other structures that may link cellular function to nuclear plasticity/deformation are being searched for^9–13^. Current methods to investigate the cell nucleus as a mechanosensor aim to control nuclear deformation, for example by micropipette aspiration, nanoindentation and microfluidic flow^10,14,15^. Other techniques control cell adhesion on novel materials, either by modifying the material’s stiffness or by patterning the substrate with engineered micro/nanoscale features such as pits, protrusions, channels, and pillars^16,17^. However, these substrates do not surround the cells but stand as an underlying support; thus, they are not fully able to mimic the architectural cue that regulates the fate of stem cells in situ*,* i.e. their three-dimensional (3D) spatial organisation^18^.

The innovative approach of our study is to use a scaffold enabling the cells to self-organize into a three-dimensional configuration allowing a more realistic study of the nuclear import flows than in any 2D cell adhesion configuration. This scaffold is a micro-fabricated substrate, produced by two-photon polymerization technique of a biocompatible, inert and mechanically stable photoresin^19^. Thanks to this optical lithographic technique, it is possible to create three-dimensional scaffolds with a resolution up to 100 nm. In our case, the scaffolds (30 μm high and 450 μm × 450 μm in transverse dimensions) are lattice of interconnected lines forming pores of graduated size, spaced between 10 and 30 μm transversely and with a uniform spacing of 15 μm vertically. From now, in this paper, these substrates will be called “Nichoid” for convenience.

Despite today, as demonstrated from the research of Discher and Engler groups^2^, it is well known that substrate stiffness correlates with stem cell fate, another factor influencing cellular functionality is the architecture of the surrounding environment. Bao et al., for example, demonstrated that substrate architecture plays an important role in generating specific cell adhesion configuration and in remodelling actin cytoskeletal organization or the shape of the cell nucleus^20^. Another example is the Nichoid, which is able to modulate cell morphology and gene activation in several types of cells, based on purely architectural cues^19,21,22^. In this paper, our modelling approach, based on experimental results, aims at providing a mechanistic interpretation of nucleocytoplasmic protein transport and protein localization. Our interesting findings are that in MSCs grown inside the Nichoid, a transcriptional activator of myogenesis fused with a GFP (MyoD-GFP) is retained in the cytoplasm and that its nuclear import flow is reduced. In addition, this reduction occurs also for GFP proteins that translocate into the cell nucleus by passive diffusion. Finally, our results suggest that cell morphology modulates the protein nuclear import through a partition of the nuclear envelope (NE) surface.

## Results

### Spatial organization of focal adhesions (FA) and actin filaments in MSCs adhering to the Nichoid artificial niche

**Figure 1:**
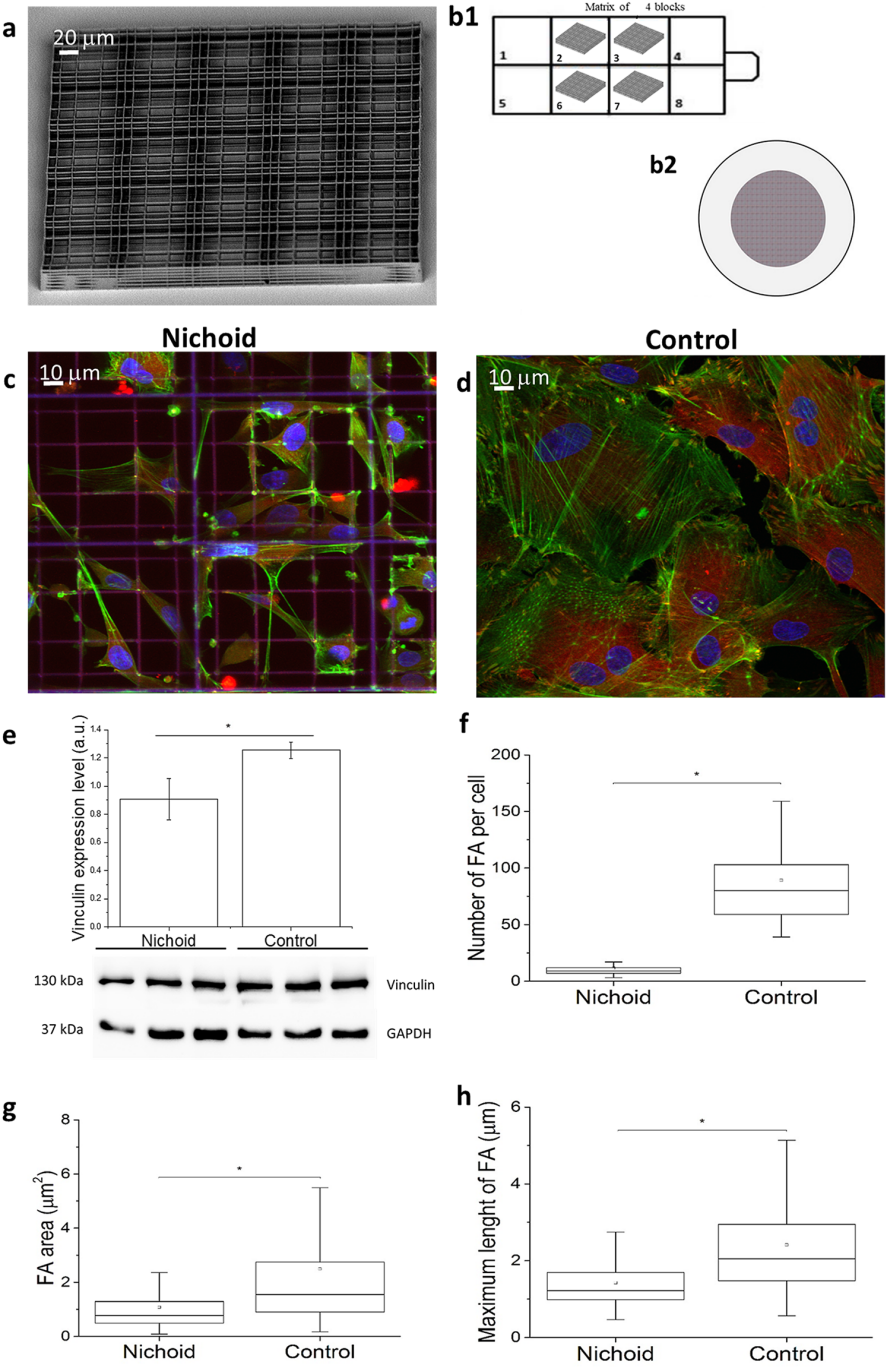
Spatial organization of focal adhesions in MSC adhering to the Nichoid artificial niche. (**a, b**) SEM images of the Nichoid microstructured module cell scaffold and schematic representation of the samples produced (**b**): **b1** glass bottom Nunc Lab-Tek-8-chamber wells and (**b2**) 1.2 cm diameter glass culture coverslip. The software used to create the image b1 and b2 are Microsoft PowerPoint 2018 (https://products.office.com/it-it/powerpoint) and SolidWorks Education Edition 2020 (https://www.solidworks.it/sw/education/mechanical-engineering-student-software.htm). (**c, d**) Immunostaining of nuclei (blue), actin (green) and vinculin (red) in cell grown into the Nichoid (**c**) and on glass flat substrate (**d**). (**e**) Vinculin expression level calculated by western blot technique: actin production is reduced in cells grown into the Nichoid. (**f**) Number of focal adhesions per cell. Spread cells present a high number of traction forces sites respect to the cells grown into the Nichoid. (**g**) Focal adhesion area. The Control shows the presence of big focal adhesions respect to the cell into the Nichoid. (**h**) Focal adhesions length. The Control presents a big number of mature focal adhesions (2 µm < length < 20 µm). Graphs statistics: Mann–Whitney test. *Correspond to p < 0.05.

The Nichoid is a miniaturised scaffold made of repeated units consisting of a 3D grid with micrometric pores surrounded by vertical walls (Fig. 1a,b1,b2). Immunofluorescence images show the cell morphology and cellular architecture of MSCs grown in the Nichoid with respect to cells grown on glass flat substrate, which we consider as the experimental 2D control (Control). Figure 1c,d presents z-projections of immunofluorescence acquisitions showing the cell nuclei (blue), actin filaments (green) and vinculin (red). As shown in Fig. 1c, the cytoskeletal organization of MSCs in the Nichoid prevails at the cell edge, with the formation of focal adhesions, where they attach to the substrate. Instead, in spread MSCs grown on the bi-dimensional control (Fig. 1d), the cytoskeleton is highly organized, with several sharp filaments arranged into the cytosol. Mature FAs are displaced at the edge of the cells and smaller FAs are visible at the bottom of the plasmatic membrane, around the nuclei.

**Figure 2:**
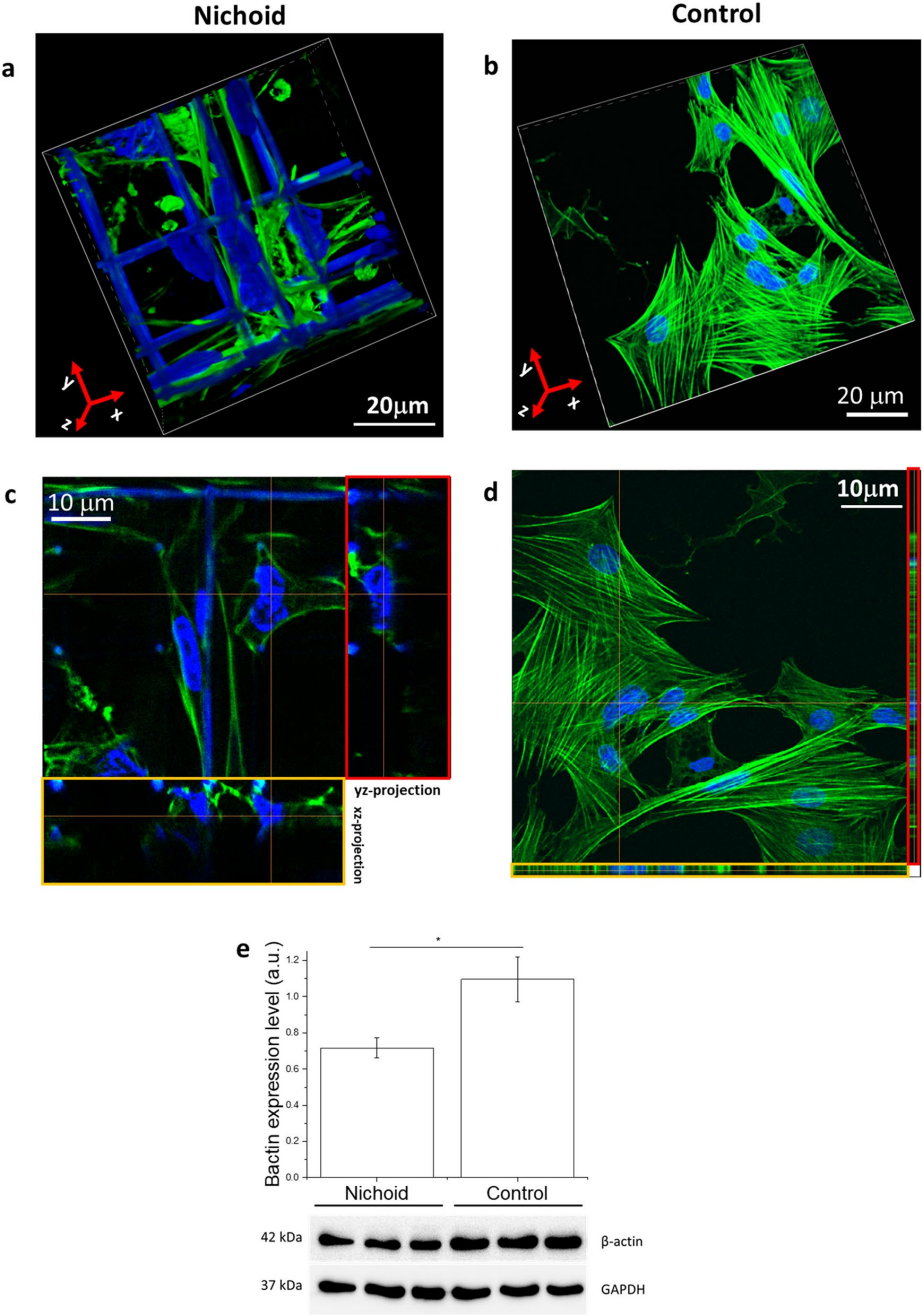
Spatial organization of actin filaments in MSCs adhering to the Nichoid artificial niche. (**a,b**) 3D reconstruction from confocal acquisitions of cells grown into the Nichoid (**a**) and on the Control (**b**). In green are identified actin fibers and in blue the cell nuclei. The SZ2080 photopolymer, used to fabricate the niche, is fluorescent at all excitation wavelengths, including at 405 nm that is the excitation wavelength of the fluorescent dye used to label the nuclear DNA. (**c,d**) Orthogonal (yellow ROIs) and sagittal (red ROIs) projections of MSCs grown into the Nichoid (**c**) and on a flat control substrate (**d**). (**e**) Actin expression level calculated by western blot. Statistics: Mann–Whitney test. *Corresponds to p < 0.05.

**Figure 3:**
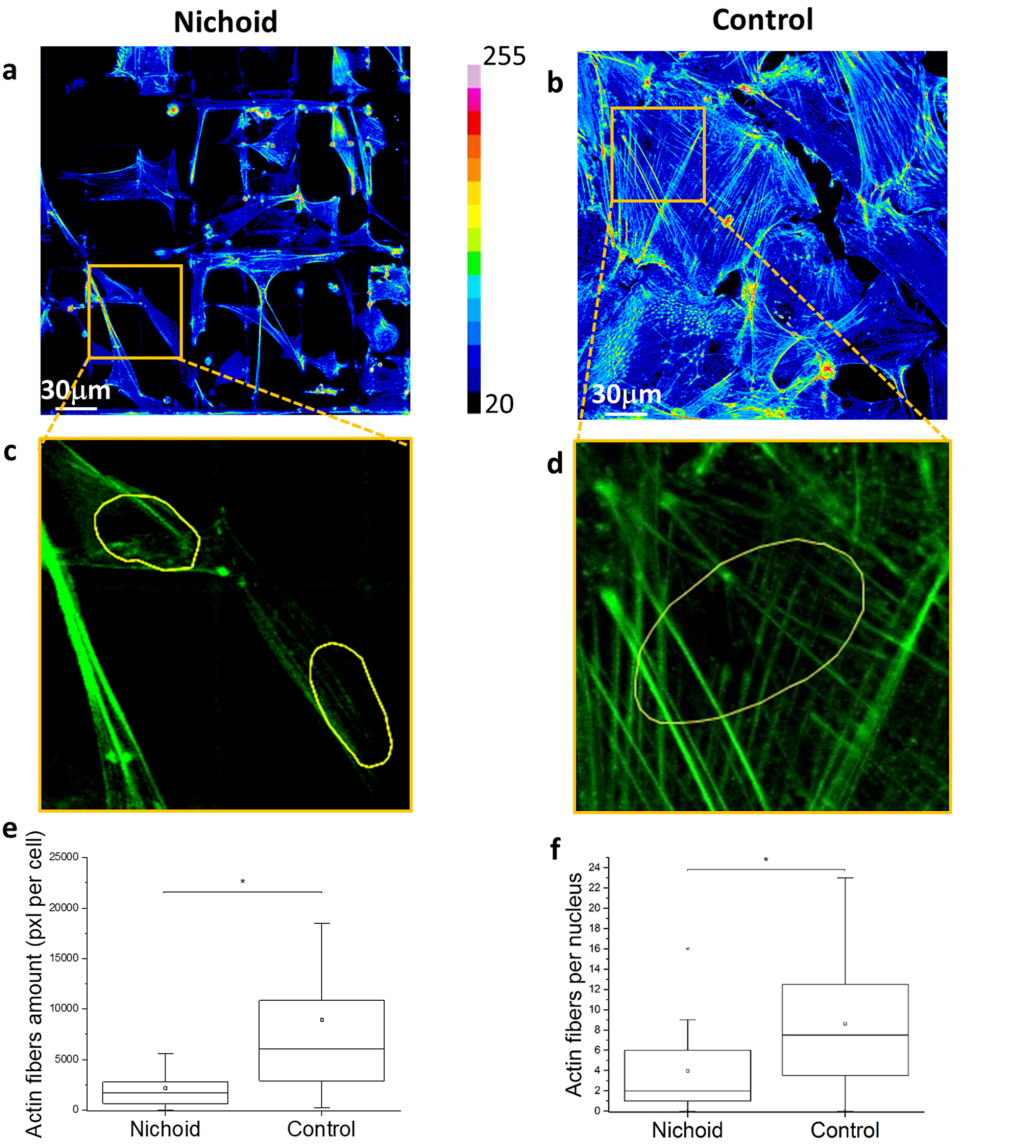
Spatial organization of actin filaments in MSCs adhering to the Nichoid artificial niche. (**a,b**) Representation of the actin fiber distribution in MSCs by using the pixel intensity color scale. (**c,d**) Representative images of actin fibers adjacent to the cell nuclei (represented by the yellow ROI). (**e,f**) Quantification of the number of actin fibers per cells and the number of actin fibers. Graphs (**e**) and (**f**) statistics: Mann–Whitney test. *Corresponds to p < 0.05.

Western blot analysis of the FAs (Fig. 1e) reveals that cells produced a significantly higher level of vinculin protein in the 2D-Control. Full length gels images are shown in Supplementary Fig. 1a,b. Fluorescence microscopy images show that these proteins are used to form visible clusters of large and mature FAs (Fig. 1d). In the Nichoid instead there are less FAs, whose length does not exceed 2 µm, which is precisely the transverse size of the trusses section forming the structure of the Nichoid (Fig. 1f–h). The formation of FAs is related to the force transmitted from the environment to the cell nucleus via actin filaments. In the Nichoid the cytoskeleton is composed of a cortical actin network, whereas long and thick actin filaments are mostly present in the Control, where cells assume a flattened and very thin “spread” shape with respect to cells adhering to the Nichoid, which are arranged in a fully 3D spatial configuration. Figure 2a,b report a 3D reconstruction of the nucleus and the cytoskeleton of MSCs grown in the Nichoid and on 2D-Controls, respectively. To better visualize actin organization, we report the orthogonal (yellow ROIs) and sagittal (red ROIs) projection of MSCs grown into the Nichoid (Fig. 2c) and on Control (Fig. 2d). All these figures effectively show that within the cells grown in 3D there are very few actin filaments and that they are spaced from the cell nucleus with respect to the cells grown in the control. To quantify the expression level of actin proteins we used western blot technique. Full length gels images are shown in Supplementary Fig. 1c,d. Figure 2e confirms the idea that cell production of actin proteins is significantly reduced in cells grown in the Nichoid compared to the 2D Control. To identify thick stress fibers, we produce z-projection images of actin cytoskeleton. Figure 3a,b show a color map of actin cytoskeleton intensity: the thicker are the actin fibers, the brighter is the fluorescence emission from the Phalloidin–FITC dye. These images qualitatively suggest the location where the force exerted by cytoskeleton fibers can be more intense: in the Nichoid, there are bright bundles of actin only at the cortical actin level. Figure 3c,d are zooms of the previous images, to better visualize the actin fibers organization around the cell nucleus: from the images it is observable that in the case of the Nichoid, very few actin fibers are present in a neighborhood of the nucleus. Finally, Fig. 3e,f show the actin fiber amount, which is known to correlate with force transmission^2–6,20^ (compression or pushing) to the cell nucleus. These results suggest that the nucleus in spread cells is subjected to a greater stress and may therefore become more deformed than in cells grown in the Nichoid.

### Spatial organization of cell morphology within the Nichoid artificial niche

**Figure 4:**
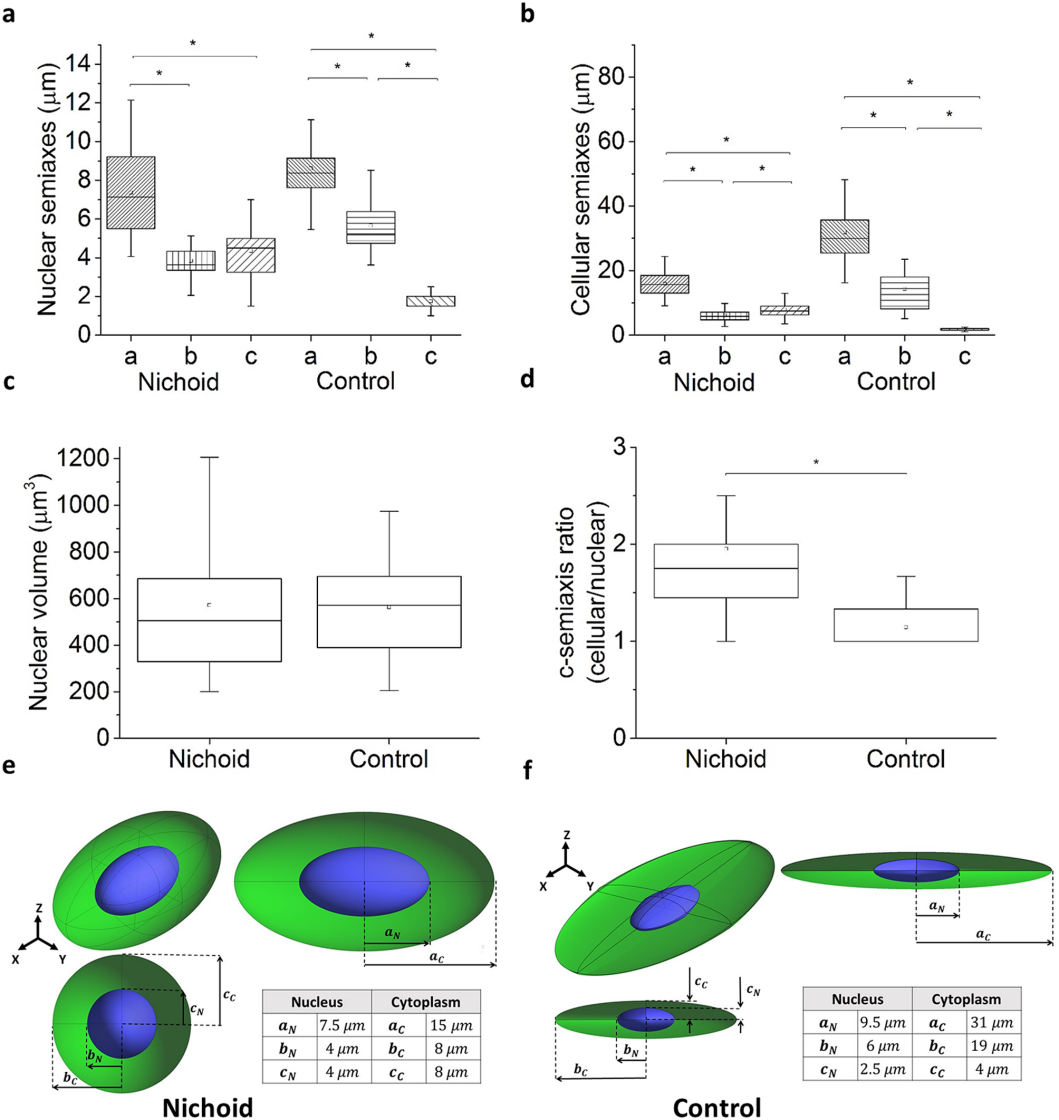
Spatial organization of cell morphology within the Nichoid artificial niche. (**a**) Nuclear semiaxes length calculated by fitting the xy, xz projection of the nuclei with an ellipse. The lengths show that the nucleus of MSCs grown into the Nichoid is modeled with a prolate ellipsoid and that the cells grown on the Control are scalene ellipsoid. (**b**) Cellular semiaxes length calculated inscribing the cell with an ellipsoid. The graph shows that the Control cells are very thin and elongate. (**c**) Nuclear volume calculates as sum of the image’s voxel. (**d**) Ratio of the cell semiaxis length divided by the nuclear semiaxis length. The graph shows that on the control the cell and the nuclear thickness are equal. (**e, f**) 3D reconstruction of nuclei (blue) of MSCs grown in the 3D-Nichoid and on the 2D-Control and semi-axes data used for the computational modelling. The software used to create the images is Ansys 2019 R2 (www.ansys.com). Graphs (**a**) and (**b)** statistics: Kruskal–Wallis multicomparison test. Graphs **c** and **d** statistics: Mann–Whitney test. *Corresponds to p < 0.05.

To evaluate if actin organization affects cell morphology, we stained the cell cytosol and the cell nucleus with two permeant and viable cell fluorescent dyes and performed z-stack acquisition with a confocal microscope. Parameters such as semi-axes, volume and distance of mass centres were used to characterize the shapes of the cell and the nucleus. Images suggest that the nucleus can be very well described and represented by an ellipsoid in both cell configurations: for MSCs grown in the Nichoid, it is a prolate spheroid, whereas the ellipsoid is tri-axial and very elongated in the *xy*-plane for the Control. On the other hand, in both conditions (the Nichoid and the Control) the cells present a star-like shape, which is very difficult to reconstruct in a computational model. Therefore, it was chosen to represent the cellular cytosol with an ellipsoid with volume equal to the average cellular volume. Experimental data of the three ellipsoid semi-axes are reported in Fig. 4a,b. Figure 4c,d reports the nuclear volume (that is the same for cells grown into the Nichoid and on the Control) and the ratio between the nuclear and the cellular semi-axes. These data confirm the observation that the nuclear shape of cells grown on Control is a very thin disk, whereas cells grown into the Nichoid are roundish, like a prolate ellipsoid. To assess if the nuclear morphology of spread cells is due to the tension of actin cytoskeleton, we inhibited actin polymerization and disrupted cytoskeleton organization by using the Cytochalasin-D drug. Our results confirm that the nuclear shape is governed by forces associated with actin cytoskeleton organization because its depolymerization makes the nucleus roundish (Supplementary Fig. 2a). In the Nichoid, the cells occupy the pores of the structure, with anchoring points often coinciding with the vertices where there is more material. Figure 4d and Supplementary Fig. 2b show the ratio between the semi-axes of the cell and nucleus to highlight the amount of cytoplasm surrounding the nucleus of MSCs grown in the Nichoid scaffold, whereas the nuclear volume to surface ratio, of which has not been observed significant differences, is reported in Supplementary Fig. 2c. In both cellular configurations, the nuclear volume and the surface are calculated as described in “Materials and Methods”.

Finally, to understand how the organization of the cell cytoskeleton affects cell morphology, the distance between the center of mass of the whole cell and its nucleus was calculated. As shown in Supplementary Fig. 2d,e, while the 3D structure does not significantly affect the relative position between the whole cell and its nucleus in the cell equatorial plane, along the z direction the Nichoid allows a higher mobility of the cell nucleus. To conclude the paragraph, the representation of the cell shape and rounded values of the semiaxes length used in the computational model are reported in Fig. 4e,f.

### Nucleo-cytoplasmic fluxes of proteins inside the Nichoid artificial niche

**Figure 5:**
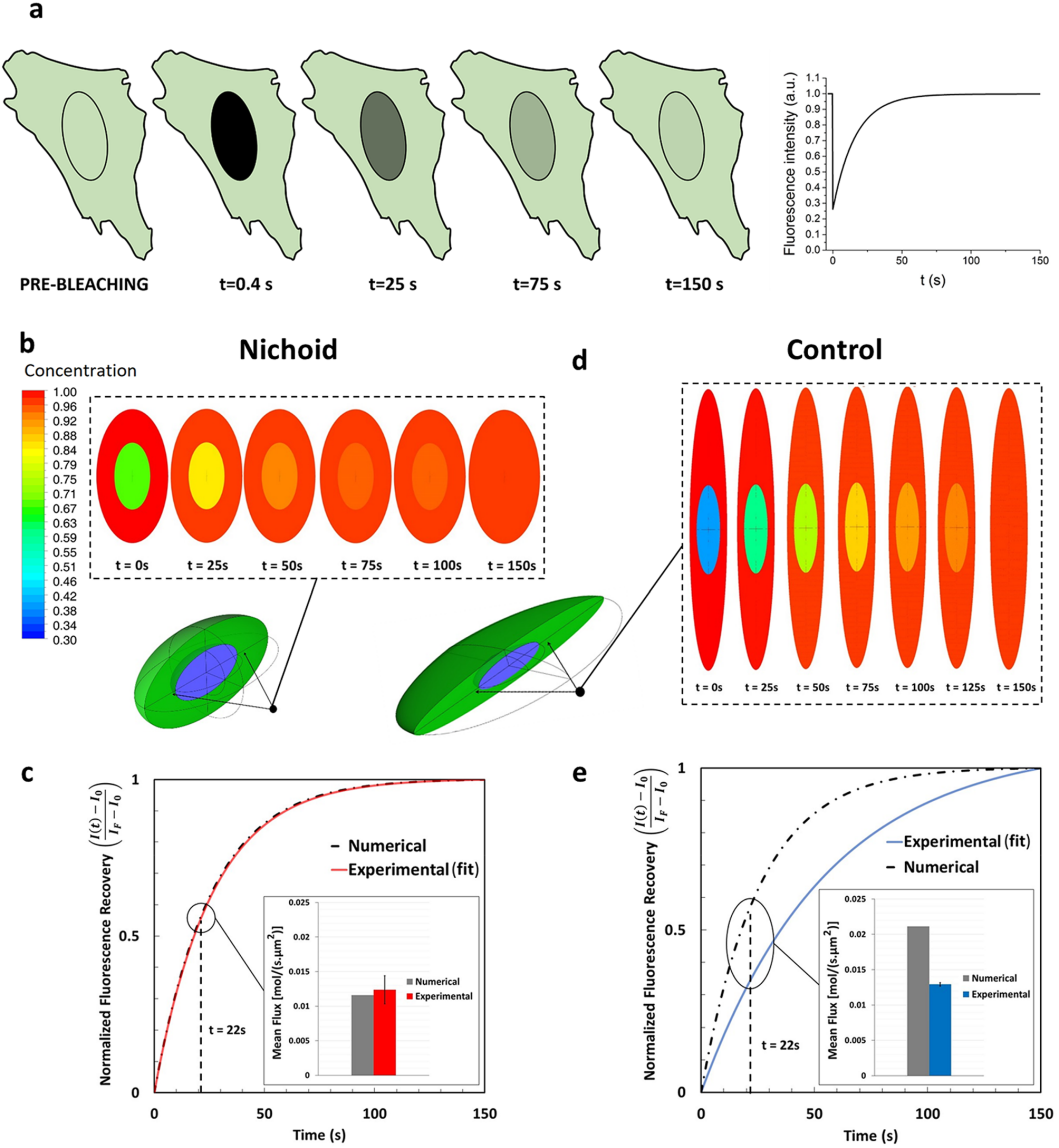
Passive Nucleo-cytoplasmic protein fluxes of proteins within the Nichoid artificial niche. (**a**) Representative sketch of FRAP experiment to study fluorescent protein translocation into the cell nucleus and a representative plot of the fluorescence intensity recovery curve. The software used to create the images are Microsoft PowerPoint 2018 (https://products.office.com/it-it/powerpoint) and OriginPro 2019 (https://www.originlab.com/origin). (**b**) Contour map of GFP concentration on xz-plane at different time points for cells grown into the Nichoid (from numerical simulation). The software used to create the images is Ansys 2019 R2 (www.ansys.com) (**c**) Comparison of recovery curves and mean fluxes at 22 s after photobleaching for cells grown in the Nichoid: numerical simulation (black dotted curve), FRAP experiments (red curve). (**d**) Contour map of GFP concentration on xz-plane at different time points for cell on Control cells (from numerical simulation). The software used to create the images is Ansys 2019 R2 (www.ansys.com) (**e**) Comparison of recovery curves and mean fluxes at 22 s after photobleaching for cells grown on a flat control substrate: numerical simulation (black dot), FRAP experiments (blue). The vertical axis of the graphs (**d**) and (**e**) shows the normalized fluorescence intensity for FRAP and normalized concentration for the numerical simulation.

**Figure 6:**
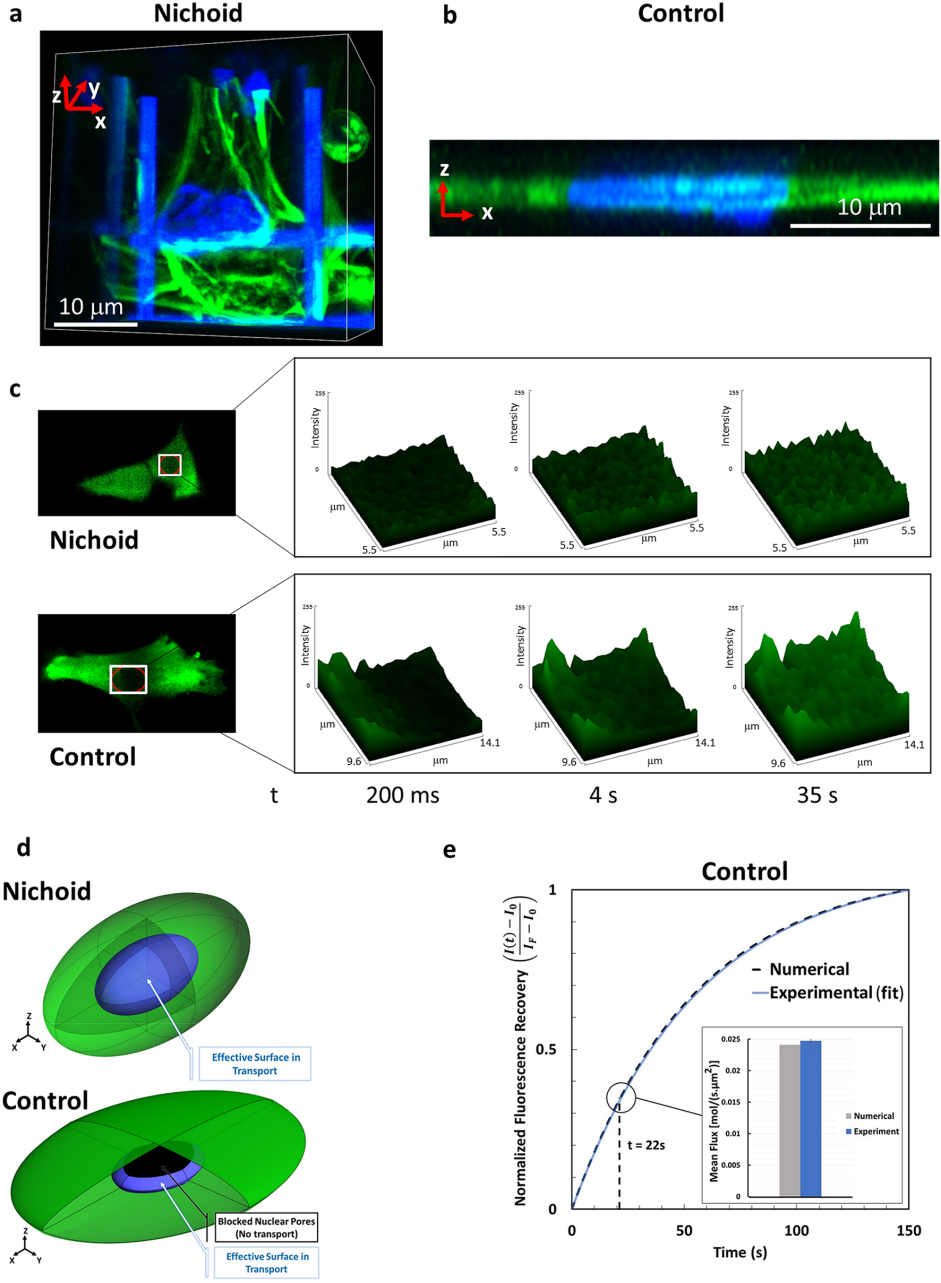
Effective NE surface for nucleo-cytoplasmic protein fluxes. (**a**) 3D rendering of MSCs grown in the Nichoid. Actin fibers are in green and the cell nuclei are in blue. The cell nucleus is surrounded by the cell cytosol in each direction. (**b**) 3D rendering of z-stack images: actin (green) and nuclei (blue). The image shows that on a 2D flat control substrate, MSCs are very thin and the volume of the cell cytosol at the top and the bottom of the nucleus is negligible. (**c**) Collection of explanatory images of the first seconds after the photobleaching. These results show that, fluorescence recovery starts at the nuclear periphery and gradually diffuses towards the nuclear centrum. In fact, the fluorescence recovery in the nuclear slice is more uniform in the Nichoid than in the Control, where a fluorescence gradient towards the center of the nucleus is clearly visible. On the left there are representative images of MSCs expressing GFP protein. The red ROI highlights the cell nucleus. The white ROI shows the rectangle on which the pixel-by-pixel fluorescence intensity plots were made. From left to right surface plots are shown at different times: 200 ms, 4 s, 35 s. (**d**) Scheme of the nuclear surface (in blue) exposed for protein transport in MSCs grown into the Nichoid (up) and on a flat glass substrate (down). The software used to create the images is Ansys 2019 R2 (www.ansys.com). (**e**) Comparison of recovery curve and mean flux within first 22 s after photobleaching for cells on a flat control substrate considering the effective surface of transport: numerical simulation (gray), FRAP experiments (blue).

**Figure 7:**
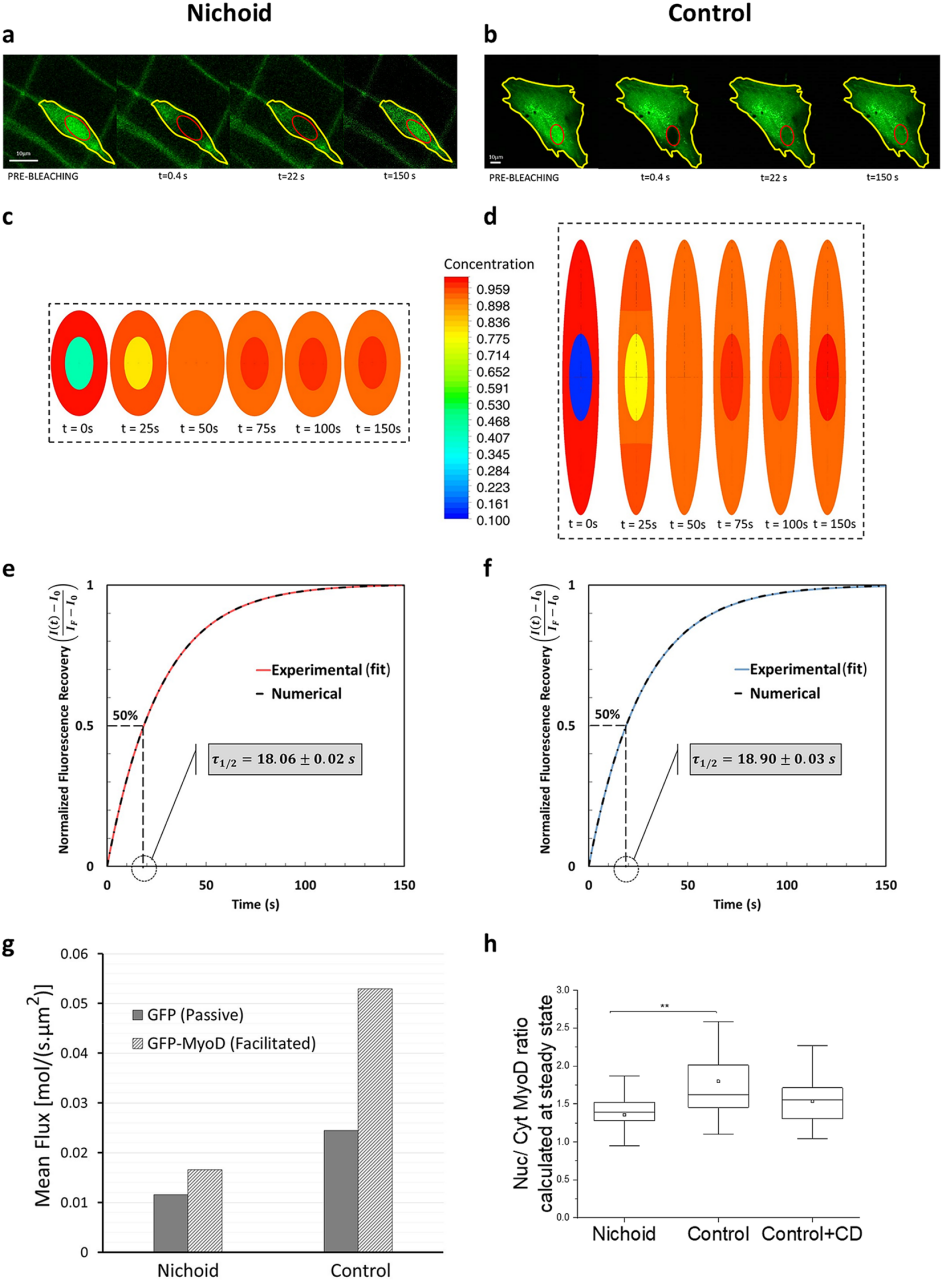
Facilitated transport through the MSC pore complexes. (**a,b**) Representative FRAP experiment on MSCs expressing GFP-MyoD protein, grown in the Nichoid (**a**) and on the control substrate (**b**). (**c**) Contour map of GFP-MyoD concentration on xz-plane at different time points for cells grown in the Nichoid (from numerical simulation). (**d**) Contour map of GFP-MyoD concentration on xz-plane at different times for cells grown on a flat control substrate (from numerical simulation). The software used to create the images (**c**) and (**d**) is Ansys 2019 R2 (www.ansys.com). (**e**) Comparison of fluorescence recovery curve for cells in the Nichoid: numerical simulation (gray), FRAP experiments (red). (**f**) Comparison of fluorescence recovery curve for Control cells; numerical simulation (gray), FRAP experiments (blue). The vertical axis of the graphs e and f shows the normalized fluorescence intensity for FRAP and the normalized concentration for the numerical simulation; the grey box inside these figures shows the half-life recovery time. (**g**) Comparison of mean fluxes (passive and facilitated) calculated by computational modelling, within the first 22 s after photobleaching for cells in the Nichoid and on the Control. (**h**) GFP-MyoD concentration in the cell nucleus at steady state conditions. The nuclear concentration in cells with thin disk-like shape is bigger than in MSCs with roundish nuclei. Graph (**h**) statistics: Kruskal–Wallis multicomparison test. *Corresponds to p < 0.05.

Fluorescence recovery after photobleaching (FRAP) experiments were used to evaluate the GFP and the MyoD-GFP nucleocytoplasmic translocation. A representative scheme of FRAP technique is shown in Fig. 5a. To characterize the processes and to evaluate the amount of protein involved, the half-time of the recovery and the protein fluxes were calculated. The results are summarized in Figs. 5, 6 and 7. The temporal profile of the recovery curve describes the kinetics of the process, from which we can calculate the flux of proteins that cross the NE using Eq. (6). To study the passive diffusion across the nuclear pore complexes (NPCs), we used the inert fluorescent protein GFP with molecular weight 27 kDa, which is far lower than the threshold known for facilitated transport^23,24^. Raw data are shown in Supplementary Fig. 2a. Figure 5, instead, reports the fitting of the experimental results. To compare the results of our numerical model with those obtained from FRAP experiments, the idealized geometries of cells in the two conditions, Nichoid and Control, were generated (Fig. 4e,f). In our model, the NE is considered as a membrane with a non-uniform pre-defined permeability; the value of the permeability can be assigned to the NE membrane through the model setup. Figure 5b represents time-dependent contour plots of concentration within the cell in the *xz*-plane obtained from the numerical simulation for cells grown in the Nichoid, in which the numerical results match the experimental data. In fact, the corresponding recovery curve of GFP in the nucleus is plotted in Fig. 5d and compared with the fit of experimental results, showing an excellent agreement. The figure also shows a comparison of the average flux of GFPs at 22 s after bleaching, highlighting that the two results are similar. Instead, Fig. 5c shows calculated time-dependent contour plots of concentration for cells grown on the Control using the same value of nuclear permeability as the cells in the Nichoid and Fig. 5e compares the recovery curve predicted by simulation with the fit of the experimentally measured curve: in this case, the numerical simulation predicts a higher flux and faster recovery than that measured by FRAP. It is therefore mandatory to modify the simulation model to fit experimental data.

This inconsistency can be explained by the fact that NPC contributes to GFP translocation if two conditions are met: first, the pore complex is open, and second, the resident GFP in the cytoplasm has access to the inlet of the pore complex. The 3D reconstruction of MSCs grown in the Nichoid and on the Control (Fig. 2) together with the morphological MSCs analysis (Fig. 4) suggest a different accessibility of the GFP to NPCs due to the cell morphology. This is supported by the resliced of the *z*-stack acquisition along the *xz*-plane (Fig. 6a,b) where there is a noticeable difference in the cytoplasm shape and actin organization around the cell nucleus. The cytoplasm occupies all the space around the nucleus of the cell located in the Nichoid. On the other hand, in MSCs grown on the Control, the plasma membrane and the nucleus of the cell are at an extremely limited distance, less than our microscope resolution, suggesting a limited transport of any molecule. Further experimental confirmations, for instance by super resolution microscopy together with molecular dynamic simulation, are required to provide important insights into this observation. However, to support our hypothesis, we show in Fig. 6c a collection of explanatory images. In fact, the recovery of fluorescence in the nuclear slice is more uniform in the Nichoid than in the Control, where a fluorescence gradient towards the center of the nucleus is clearly visible (see Fig. 6c, bottom pannel). These results suggest on the one hand that, fluorescence recovery starts at the nuclear periphery and gradually diffuses towards the nuclear centrum. On the other, they support the hypothesis of a larger exposed surface of the nuclear envelop to the cytoplasm in the Nichoid as compared to the Control, that will favor a much more uniform fluorescence recovery in the first case.

Hence, we continue with our conjecture to prove that our hypothesis is plausible. In fact, considering the homogeneity of both nuclear pores distribution and the density of fluorescent protein, the extremely small portion of volume available for protein diffusion, that the diffusion in the cytoplasm is much faster than nucleocytoplasmic transport (and therefore the second is a disadvantaged process), then protein transport can be neglected. Hence, we suggest that in the Control, not all the surface of the NE contributes with the same effectiveness to the transport of the GFP. In order to account for these observations, in our numerical simulation for flattened cells, we divided the nuclear surface in two parts: i) the first through which effective transport occurs, and ii) a second one in which transport is prevented (Fig. 6d). Therefore, our approach toward addressing this observation is to use the numerical simulation to calculate the fraction of the nuclear envelope that should be blocked in order to reproduce the experimental results obtained by FRAP technique recovery curve. The procedure to calculate the blocked surface was as follow: in Fig. 5e the numerical recovery curve corresponding to the case in which the entire surface of the NE is considered permeable for the transport is presented. In this case, the numerical results do not match the experiments. Then, we systematically decreased the permeable portion of the NE and compared the recovery curve from numerical simulation with the experimental one. The final result is presented in the Fig. 6e: it shows the same experimental data of the Fig. 5e, but the numerical recovery curve has changed due to the reduction of the effective surface in transport of the GFPs. The calculated effective surface results in a 46% of the total NE surface. Here, our first conclusion is that, for the cells grown on the stiff and flat substrate, a significant portion of the NE has a negligible contribution to the nucleo-cytoplasmic transport. Obviously, the correction of the nuclear effective surface depends on the substrate physico-chemical properties and, therefore, it must be recalculated according to the cell morphology.

The combination of the fact that the transport occurs along less than half of the nuclear surface, with the transport kinetics that has been found slightly slower $(\Delta {t}_{1/2}\approx 33\;\text{s})$ on MSCs grown on Control with respect to the cells into the Nichoid, suggests that the pores available for transport are more open. The conjecture is confirmed by the flux calculated, using Eq. (6), at 22 s after photobleaching: it indicates that the GFP influx in spread cells is 2.1-fold higher than the flux in MSCs grown in the Nichoid (inset of Fig. 6e).

To understand the effect of cell morphology on the rate of facilitated transport of large proteins, we studied the transport of GFP-MyoD, a transcription factor (fused with a GFP) implicated in the differentiation towards myogenic lineage. The total molecular weight of MYOD-GFP is around 62 kDa; it is big enough to require facilitated transport. Moreover, MyoD is known to possess the nuclear localization signal (NLS) domain that guides the facilitated transport process.

A representative nuclear fluorescence recovery, corresponding to the import of the GFP-MyoD during the FRAP experiment, is shown in Fig. 7a, b for cells grown in the Nichoid and on the Control, respectively. The fluorescence recovery starts after the photo-bleaching, slows down over time, and ends when a certain ratio of nucleus-to-cell concentration of GFP-MyoD is achieved. (Raw data of the fluorescence recovery are reported in Supplementary Fig. 3a,b). Instead, Fig. 7c,d show the protein concentration results obtained by the simulation. For the Control cells, we considered the effective nuclear surface used to reproduce the FRAP experiments with the GFP and, for the cells in the Nichoid, the entire nuclear surface. Using these parameters, we found the corresponding permeability that enabled us to well represent the experimental FRAP curve with the numerical simulation for both cases, cells in the Nichoid and on the Control substrate (Fig. 7e,f). The figures show that the half-time recovery for both configurations in the case of the facilitated transport is quite similar (${t}_{1/2}\approx 18$ s). This contrasts with the kinetics of passive transport, as shown in Fig. 5d,e, and it highlights the fact that the two processes are not equally affected by the cell morphology. The fact that the half-time in the kinetics of spread cells has shortened with respect to the passive transport equalizing the half-time of cells grown in the Nichoid suggests that the NPC permeability in facilitated transport must be higher than in the Nichoid. The calculation of GFP-MyoD translocation fluxes confirms this conjecture. In fact, the flux in spread MSCs is 3.1 times higher than in cells grown in the Nichoid (Fig. 7g).

To observe if cell morphology can also affect the nuclear concentration of proteins, we calculated the fluorescence intensity of nuclear MyoD-GFP. Our results show that the morphology adopted by MSCs grown in the Nichoid changes the balance ratio of the transcription factor concentration into the nucleus, which is 20% less than the concentration in the Control (Fig. 7h). Finally, in order to reduce the deformation of the nucleus in the cells grown on a stiff and flat substrate, we used Cytochalasin-D drug. Its depolymerizing action of the actin fibers reduce the tension on the cell nucleus, which assumes a roundish shape. In fact, the results obtained on the nuclear geometry showed that, using Cytochalasin-D, the nuclear shape becomes roundish and tends to be more similar, but not exactly the same, to the nuclear shape of cells grown in the Nichoid. In the case of Cytochalasin-D treated cells, the Myod-GFP nuclear concentration is reduced around 10% respect to the Control, placing itself at an intermediate concentration between the Control and Nichoid samples.

**Figure 8:**
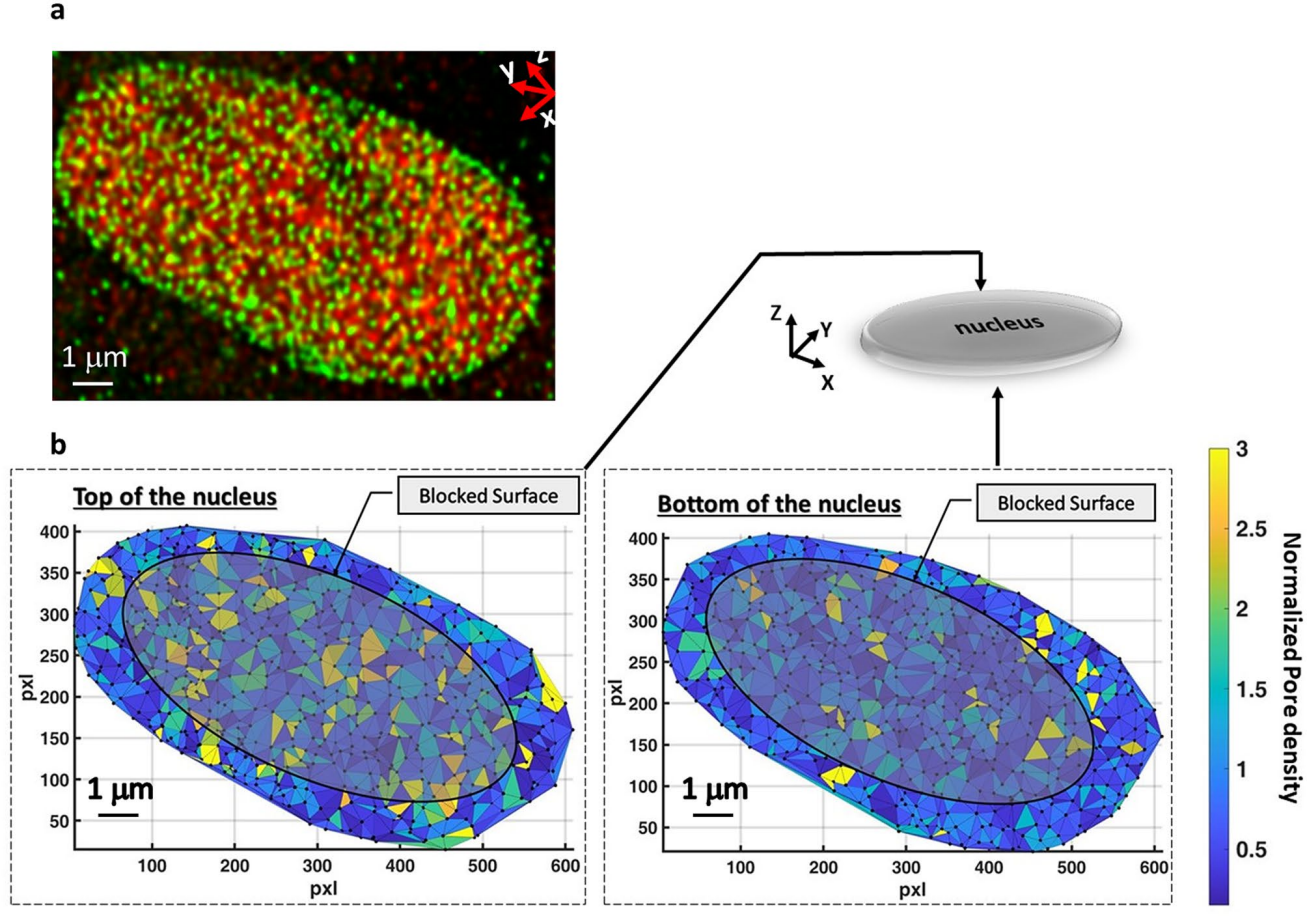
Mean nuclear flux and pore distribution. (**a**) 3D reconstruction of confocal z stack images. In green is visible the nuclear pore distribution and in red the DNA (Nup153 protein and H3K4me3 staining respectively). (**b**) Superimposition of the NPC nuclear localization and the transport-blocked surface; the surface is reconstructed by Delaunay triangulation method and the color shows the normalized NPC density at each triangle elements. The software used to create the two images is Matlab R2019b (https://it.mathworks.com/).

However, from a mechanistic point of view, the higher permeability may be associated with either a larger number of pores per unit surface, a higher permeability of each single pore, or a combination of both. To examine the first possibility, we stain NPCs with the Nup153 antibody and the cell nucleus (by using the H3k4me3 antibody) and imaged them using confocal microscopy. A representative image of one MSC grown on the Control is reported in Fig. 8a. Already qualitatively Fig. 8a suggest that the distribution of NPCs as a function of the deformation of the nucleus is quite homogeneous. the exact spatial position of the NPCs was extracted with the software Huygens to create Fig. 8b, where the 3D localization of NPCs is shown together with the nuclear effective and blocked surface. This schematic figure visualizes the distribution of NPCs in the entire nuclear membrane. The color of each triangle element connecting three NPCs shows the normalized pore density which is varying locally between 0.15 and 3 in the NE (the density is normalized by the mean value over the whole surface of the NE). In our samples any particular patterns in NPCs density was observed. Therefore, the possibility that the higher permeability of the nuclear surface exposed to the cytosol (effective surface) is due to the higher density of NPCs at that region appears to be not supported by our analysis. On the contrary, the hypothesis that the increase in permeability obeys to an opening of the nuclear pores appears reasonable.

## Discussion

Mechanical and biochemical factors regulate stem cell renewal or differentiation. Thanks to integrin-mediated FAs, cells can anchor to the substrate, sense the surrounding microenvironment, and provide a mechanism through which environmental mechanical forces determine the stem cell function and fate. Extracellular cues are thus translated to the nucleus via actin fibers, triggering gene expression, sensing environmental forces, internalizing membrane vesicles, providing internal forces, moving, and dividing cells. Actin therefore contributes to fundamental biological processes^25^. Understanding how biophysical cues in the Nichoid regulate cell function is important, since it leads to a much better understanding of how cells develop and maintain their distinctive properties. It also enables us to understand how to design new scaffolds and what features need to be considered in devices for tissue regeneration. Micropatterning techniques and substrate stiffness regulation are commonly used to control cell shape and behaviour, although 2D cell cultures are not completely representative of the in vivo cell phenotype. Researchers have recently been investigating how the 3D environment can influence cell morphology and cell function^20^.

We employed a microfabricated 3D culture substrate, the Nichoid, which modulates cell morphology and gene activation based on architectural cues that condition cell adhesion^19,21,22,26^. As 2D Control substrates, we used glass coverslips, which underwent the same treatment steps of the Nichoid samples, except for the exposure to the polymerization laser beam Our previous findings using the Nichoid^22,26^ and the new results obtained here using Cytochalasin-D to inhibit actin polymerization, suggest that 3D architectural cues of the Nichoid determine the spatial configuration of the forces that guide MSC morphology and functionality. In cells, force transmission depends on the FA strength, which is related to the FA dimension. There are small initial contacts mainly located at the cell edge, the so-called “Dot” contacts, and there are mature FAs, the “Dash” contacts, associated with the cytoskeletal actin bundles^27^. Our analysis on vinculin expression and localization reveals that mature FAs only occur in the Control cells (Fig. 1), suggesting that the forces transmitted to the nucleus in MSCs grown in the Nichoid are weaker than those transmitted in spread cells (Fig. 2). The actin staining also confirms the hypothesis suggested by different research groups^28–30^ that there is a mechanism by which the shape of the nucleus can be controlled by the underlying distribution of FAs. In our experiments, MSCs grown in the Nichoid lack the perinuclear actin cap, contrary to cells grown on the Control (Figs. 2, 3, 5). This structure may be associated with the observed deformation of the nucleus since it pushes and compresses the nucleus toward the bottom of the cell, preventing the nucleus from bulging against the cell membrane while providing the observed disk-like shape.

For MSCs grown in the Nichoid, the role of the actin fibers is mostly associated with the creation of a structure, the actin cortex, that allows the cells to grow in a 3D environment, which offers few adhesion points and therefore provides few sites for the transmission of internal forces from the environment to the nucleus. Other researchers have obtained a similar result using substrates with a different stiffness, diversifying the cell volume, depleting FA proteins or affecting the MAPK signalling^31–33^. They have thus confirmed that the nucleo-cytoplasmic translocation of proteins is mediated by forces transmitted to the nucleus. By disrupting the cytoskeletal organization, using Cytochalasin-D drug, we demonstrated that the actin cap squeezes the nucleus, inducing the thin disk-like shape. This modulation of the nuclear morphology affects the overall nuclear permeability, at least for the inert GFP protein and for the transcription factor MyoD fused with a GFP (Figs. 5, 6, 7). As the observations regarding cells grown on a flat substrate show, the bottom of the nucleus is in contact with the substrate below, while the top of the nucleus is in close contact with the perinuclear cap and the cell membrane, leaving almost no space for the cytoplasm (Fig. 6). It has been reported that nuclear stiffness and intracellular forces control the maximum height of the cell on a flat substrate^34^. Our work suggests that the nuclear shape and the actin organization can also alter the accessibility of the NPCs to proteins translocation, hence changing the overall nuclear permeability. In fact, our numerical simulation together with the experimental measurements show that for Control cells, the mean flux of the GFP and GFP-MyoD proteins through the effective nuclear surface is higher than that in cells grown in the Nichoid; this difference is magnified in the case of the transcription factors MyoD (Fig. 7).

Our results agree with previous published research studies. For example, in 2013, using a combination of theory, numerical simulations, and experiments, Rangamani et al*.*^33^ suggest that local curvature of the cell modulates biochemical signaling pathways due to an accumulation of activated receptors at regions of higher curvature with increasing cell eccentricity. Here, we demonstrate that in a 3D culture system, which is more similar to the physiological one, the MyoD concentration in the nucleus is lower than that obtained in a standard (two-dimensional) culture system (Fig. 7). This fact may occur due to a modulation of the nuclear permeability through either a modification of the number of pores per unit surface, a higher NPC permeability, or a combination of both. Figure 8 denies the hypothesis of a significant NPC redistribution as a consequence of nuclear deformation. Therefore, we can assume that the NPC distribution is quite homogeneous, and that the modulation of the nuclear permeability is guided by the opening of the pores. Other researchers agree on the existence of this mechanical phenomenon^31^ and our results regarding the flux of proteins entering in the cell nucleus in the two nuclear conformation together with the reduction of the NPCs amount, due to the effective surface in the deformed samples, suggest an opening of the NPCs located at higher nuclear curvature.

However, it should be remembered that this is a mechanistic interpretation of a phenomenon in which other biological components will also compete. For instance, it could be that in the Control, the physiological regulators of facilitated transport, such as proteins that are in charge of the nucleus-cytoplasm shuttling of macromolecules (*i.e.* nuclear transport receptors -NTRs), are upregulated as a consequence of the 2D adhesion environment^35^. However, the time constant of the mechanical- and genetic-related changes in permeability would differ by several orders of magnitude. In the future, we aim to explore this possibility both experimentally and with a computational model at a molecular scale.

## Methods

### Microfabrication of 3D Nichoid structures

The culture substrates used as the basis for the Nichoid production were the Nunc Lab-Tek-8-chamber wells (Thermo Fisher Scientific, Italy) or glass culture coverslip 1,2 cm in diameter. In both cases the glass category is #1.5, i.e. 0.17–0.19 µm thick, with Young’s modulus of around 80 GPa. In the first case, Nichoids were fabricated in the four central wells (7 mm × 9 mm each) and spatially organized in 2 × 2 units, each of which was 450 μm × 450 μm × 30 μm in size (Fig. 1a). In the latter, a circular area with 8 mm in diameter was fully covered with Nichoids. Detail on Nichoid fabrication are reported in^36^. The Young’s modulus of the photo-polymerized Nichoid micro-lattice is two orders of magnitude less stiff than glass^22,24^.

In all experiments the Lab-Tek bottom glass or commercial glass coverslip, which have undergone all the treatment steps except the exposure to the polymerization laser beam, are considered as 2D-control sample (Control).

Scaffolds (Nichoids and Controls) were sterilized by washing three times with deionized sterile water and incubating for a few hours in ethanol 70%, followed by 1 h UV exposure and three with deionized sterile water washes.

### Cell culture and seeding

Rat-MSCs were cultured for a few passages (maximum 5) in alpha-MEM phenol red free medium supplemented with 20% fetal bovine serum, 1% L-glutamine (2 mM), penicillin (10 units/ml), and streptomycin (10 µg/ml) at 37 °C and in 5% CO_2_ (Euroclone, Italy). Culture medium was changed every 2–3 days and cells were used at passages 1–6 after thawing.

For the fluorescence microscopy experiments, 10^4^ MSCs were seeded in each well and analyzed after immunofluorescence assay, permeable fluorescent dye or transfection treatment. For the western blot analysis 15 × 10^3^ MSCs were seeded in the Nichoid substrate, while 7 × 10^3^ MSCs were seeded on glass coverslip 2 cm in diameters. Cells were grown for one week and then lysed.

### Western blot analysis

Protein lysates were extracted using a lysis buffer (50 mM tris–HCl, 1% Triton X-100, 150 mM NaCl, 0.5 mM EDTA, pH 7.4) supplemented with 1% protease inhibitor cocktail (Sigma-Aldrich). 20 μg of protein extract was separated by SDS–PAGE and transferred onto a nitrocellulose membrane (Biorad). Non-specific binding sites were blocked with 5% BSA in Tris-buffered saline buffer with 0.1% Tween 20 (TBST) for 1 h at room temperature. Membranes were incubated with specific antibodies overnight at 4 °C (anti-GAPDH 1:1000 Cell Signaling; anti-Vinculin 1:10,000 Abcam; anti-βactin 1:10,000, Sigma-Aldrich). HRP-conjugated anti-mouse (Jackson) or anti-rabbit (Santa Cruz Biotechnology) secondary antibodies were then used to incubate membranes for 1 h at room temperature. Chemiluminescent signals were detected using Immobilon Western Chemiluminescent HRP Substrate (Millipore) and the ChemiDoc Imaging System (Biorad). Signal intensity was analyzed using ImageJ software. GAPDH was used as the endogenous control and the results were expressed relative to its levels.

### Immunofluorescence assay

To visualize the organization of the actin stress fibers and the focal adhesion localization, one day after seeding, cells were fixed for 15 min in 4% paraformaldehyde, washed with phosphate buffered saline (PBS, Euroclone, Italy) and permeabilized with 0.25% Triton X-100-PBS (Sigma Aldrich, Italy). After 15 min incubation at room temperature, they were processed for indirect immunofluorescence analysis^37^.

Cells were blocked in PBS-2%bovine serum albumin (BSA, Sigma Aldrich, Italy) for 4 h; then incubated at 4 °C, over-night, with primary antibody (mouse monoclonal anti-Vinculin antibody (Sigma Aldrich, Italy), Nup153 and H3K4ME3, Abcam UK,). After three washes, samples were incubated at room temperature for 45 min with 1 μg/ml phalloidin-FITC (Sigma Aldrich, Italy), if necessary, to stain actin cytoskeleton, and/ or with 2 μg/ml secondary antibody (Alexa Fluor, Abcam, United Kingdom). Cell nuclei were stained with a 10-min incubation using 1 μg/ml of Hoechst 33342 (Thermo Fisher Scientific, Italy) in PBS. After other three washes, samples were imaged using a confocal microscopy set-up. For actin cytoskeleton, vinculin focal adhesions and nuclear morphology characterization 1 µµ step z-stack were performed with airy1 pinhole settings and 200 nm pixel dimension. A total of 21 (Nichoid) and 18 (Control) cells were analyzed. For NPC characterization 300 nm step z-stack were performed with airy 0.7 pinhole settings and 30 nm pixel dimension. Ten cells grown on controls were analyzed.

### Live cell staining

MSC nuclei were incubated with 1 μg/ml Hoecst-33342 and 0.8 µM calcein-AM fluorescent probes (Thermo Fisher Scientific, Italy) ten minutes before the experiments. After one washing step, the cell culture medium was replaced. Samples were imaged using a confocal microscopy set-up.

All the experiments were repeated independently three times for each reported dataset (Nichoid and Control). The number of cells analyzed was 32 and 28, respectively.

### 3D fluorescence images

Fixed samples maintained in PBS, and live samples maintained in fresh culture medium were imaged using a confocal microscope (Olympus Fluoview FV10i) equipped with four diode lasers (emission wavelength 405, 473, 559, 635 nm), with a 60 × water immersion objective, 1.2 N.A. (image size 212.13 × 212.13 µm^2^). The pinhole was set to 1 Airy Unit. *Z-*stack images were acquired on cells in the Nichoid microstructure (40 µm acquisition depth) and on the Control cells (10 µm acquisition depth) with a 1 µm step.

### Cellular parameter analysis

The image analysis was carried out with the open source software ImageJ (https://imagej.nih.gov/ij/index.html, USA) and the data analysis was performed with Origin Pro software. Images were converted in 8 bits.

#### Focal adhesions

The number of focal adhesions, area and maximum elongation were calculated by manually drawing the regions of interest (ROIs) around FA projecting a curved surface and comparing it to a 2D surface.

analyzed.

#### Actin fibers

Actin fibers amount per cells were calculated in few steps:

1. the production of a maximum value-z-projection image of the *z*-stacks.
2. Identifying each cell profile manually drawing a ROI on their edge.
3. Filtering the -z-projection image using as minimum threshold the 30% of the total value (pixel value below 76.5 were discarded) and producing a binary image.
4. Finally, the actin fibers amount was calculated measuring the number of white pixels per cell.

Instead, actin fibers interacting with the cell nucleus were calculated by using the same maximum value-z-projection image of the *z*-stacks. ROIs identifying the cell nuclei were manually drawn on the Hoechst33342 channel and then reported on the actin channel. The number of actin fibers crossing the nuclear ROI were manually counted.

#### Cell morphology

Samples treated with Hoechst-33342 and Calcein-AM were analyzed separately to evaluate the morphological feature of MSCs.

To evaluate the semi-axes, two orthogonal projections (*xy* and *xz*) of the *z-*stack were performed for each cell. This solution identifies the largest *xy* and *xz* nuclear and cellular sections. The semi-axes *a, b* and *c* were obtained by fitting the results with ellipses*.* The ratio between the cellular and nuclear semi-axes *c* returns the cell elongation along the *z*-axis.

The coordinates of the cellular and nuclear mass centers were calculated by drawing two ROIs around the cell in two orthogonal projection planes.

The nuclear and the cell volume were calculated manually by drawing a ROI for each cell z-stack and calculating the sum of voxels included in the ROIs.

For the Control dataset, nuclear surfaces, *S*, were calculated using the scalene ellipsoid (*a* > *b* > *c*) formula:

1 $$S=4\pi \sqrt[p]{{\left(ab\right)}^{p}{\left(ac\right)}^{p}{\left(a\right)c}^{p}}$$

where p = 1.6075.

For the Nichoid dataset, nuclear surfaces, *S,* were calculated using the prolate ellipsoid (*a* > *b* = *c*) formula:

2 $$S = 2\pi \left( {c^{2} + ac\frac{{\arccos \left( {\frac{c}{a}} \right)}}{{\sin \left( {\arccos \left( {\frac{c}{a}} \right)} \right)}}} \right)$$

### GFP-MyoD cloning

In order to create the GFP-MyoD recombinant protein, we fused MyoD onto a supernegative variant of GFP created by Lawrence and colleagues^38^. We amplified the (-30)GFP sequence from the pET-(-30)GFP-9xGGS-Cre-6xHis (Addgene, plasmid #62,372). We fused the MyoD sequence at the (-30)GFP-C terminal and inserted the recombinant sequence in the eukaryotic plasmid pcDNA3 using the NdeI and HindIII restriction sites. We used the TOP10 *E. coli* strain for GFP-MyoD cloning procedures.

### Cell transfection

In order to study the passive diffusion, cells were transfected with a plasmid coding for a standard GFP (pmaxGFP), while in order to study facilitated transport, MSCs were transfected with pcDNA3-GFP-MyoD. Transfection procedures were performed using a commercially available protocol (jetPRIME, Polyplus Transfection, USA). Briefly, a solution composed of 0.5  µg of DNA, 25 µl of jet PRIME buffer and 1.12 µl of jet PRIME reagent was prepared and kept at RT for 15 min. Cells were incubated with the transfection solution added to 400 µl of antibiotic free medium (alpha mem, 20% fetal bovine serum (FBS), 1% L-Glutamine; Euroclone, Italy). After 4 h, the solution was replaced with a complete medium^38^. Before FRAP experiments, cell nuclei were labelled with 1 µM DRAQ5 fluorescent probe (ThermoFisher, Italy).

### Fluorescence recovery after photobleaching (FRAP)

FRAP measurements were performed as described in^39,40^ with minor modifications. Briefly, images were acquired with a confocal Laser Scanning microscope (Leica SP8) equipped with an Argon laser and a white light laser, a 63X PlanApo oil-immersion objective (NA 1.4) and an incubator chamber. Image size was 256 × 256 pixels and the scan speed was 700 Hz. Pinhole size was set to reach a *z*-resolution of 3 µm. More than 20 cells per dataset (Nichoid and Control) were acquired in three different experiments.

To detect the cell nucleus and choose the best plane to perform the FRAP measurement, DRAQ5 dye was detected using 8% of the Leica White Light Laser (excitation 633 nm, emission 650–750 nm). For each cell, we identified and recorded a ROI describing the section of the nucleus for the FRAP measurements. The images were collected using, at the sample, around 15 µΩ excitation power of an Argon Laser (excitation wavelength 488 nm, emission wavelength 500–580 nm) was used. Nuclear proteins were photobleached by scanning a nuclear ROI with the 488 nm laser at full (100%) power. Three to five seconds were required to photobleach most of the nuclear fluorescence, without destroying too much of the cytosolic fluorescence, in cells spread on glass substrate. For the cells grown in the niche, in order to avoid bleaching the fluorescent proteins present in the cytoplasm, the maximum photobleaching time was in the order of hundreds of milliseconds. The plots of Fig. 6c, showing the pixel-by-pixel fluorescence intensity of a rectangular ROI circumscribing the cell nucleus during the fluorescence recovery, were created using the ImageJ "Surface Plot" plugin.

Nuclear fluorescence recovery was measured by starting a time-lapse acquisition within a few hundred milliseconds (382 ms) after the bleaching, acquiring 20 images every 191 ms and then 90 images 241 every 6 s in the case of GFP, and acquiring 200 images every 191 ms and then 100 images every 5 s to study GFP-MyoD recovery. As shown in Supplementary Fig. 3c–f the different bleaching time between cells grown in the Nichoid and on the Control is necessary to obtain a significant bleaching of the nuclear fluorescence. The bleaching depth is 0.7 ± 0.1 e 0.8 ± 0.1 for MSC grown on flat substrate and expressing GFP and MyoD-GFP proteins, respectively. In the case of Nichoid the bleaching depth is 0.5 ± 0.1 0.6 ± 0.1, respectively. During the bleaching time, it is inevitable that some fluorescent proteins into the cytoplasm will also bleached; however, as shown in Supplementary Fig. 3g,h it does not introduce considerable artefacts because the recovery of the nucleus is governed by the permeability of the NE and the transport through the NE is about three orders of magnitude slower than transport inside the cytoplasm and nucleoplasm. It means that the cytoplasm recovers in some milliseconds while transporting through the NE takes some of seconds. Therefore, the effect of NE as a membrane that hinders the recovery of the nucleus is dominant enough. The recovery of the fluorescence was evaluated for at least 9 min, which is enough time to observe a long plateau in the fluorescence intensity recovery curve. In each acquisition, the image background was subtracted from the curves.

The fluorescence signal was assumed as being proportional to the protein concentration and described by the function:

3 $$F\left(t\right)={F}^{\infty }\left(t\right)+({F}^{0}\left(t\right)-{F}^{\infty }\left(t\right)) {e}^{-\frac{t}{\mathrm{\tau}}}$$

To remove the fluorescence bleaching due to the measurements, nuclear recovery data were divided by the total cell fluorescence intensity, using an ROI drawn on the cell edge as shown in Fig. 4c.

Data of the three experiments were mediated and fitted (Origin Pro software) to a single exponential function using the following equation:

4 $$y={y}_{0}+A{e}^{-\frac{t}{\tau}}$$

where *τ* is the characteristic time of the protein translocation from cytosol to the nucleus, A is the difference between the nuclear fluorescence after bleaching and the nuclear fluorescence at the end of the recovery, corresponding to the fraction of protein involved, and y_0_ is the fluorescence measured at the end of the recovery. A characteristic parameter of exponential decay is the half-life (the time needed to reach 50% of the total amount of fluorescence) which is calculated as ${t}_{1/2}=\tau \mathrm{ln}2$.

Data after the bleaching time were considered and then normalized between 0 and 1 using the following equation:

5 $$I\left(t\right)= \frac{{I}_{cell}(t)-{I}_{0}}{{I}_{f}-{I}_{0}}$$

where ${I}_{cell}\left(t\right)$ is the recovery curve, ${I}_{0}$ is the intensity value of the nuclei after bleaching and ${I}_{f}$ is the value of the plateau.

The flux of proteins, GFP and GFP-MyoD, across the nuclear pore complexes were calculated by

6 $$\Phi \left(\tau \right)= \frac{{I}_{\tau }{V}_{nuc}}{\tau {S}_{nuc}}$$

where ${I}_{\tau }$ is the mean intensity at a delay *τ* after bleaching (chosen here as *τ* = 22 s), ${V}_{nuc}$ is the nuclear volume measured in the morphological analysis, and ${S}_{nuc}$ is the nuclear surface calculated by the Eq. (2). The MyoD protein concentration in the cell nucleus was evaluated, at steady state, by measuring the average fluorescence intensity in an ROI with 1.5  µ m radius, positioned at the nucleus center, and normalizing it by the average fluorescence intensity in ROI, with equal radius, positioned in the cytosol the furthest away from the cell nucleus.

### Statistical analyses

The experimental data were analysed by non-parametric test, such as Mann–Whitney U test, for the comparison just between two groups, and Dunn's Kruskal–Wallis Multiple Comparison test, when the groups are more than two. Associations with *p values < 0.05 were considered significant.

### Governing equations for passive transport

Passive transport of the GFP inside the cell is determined by Fick’s law of diffusion formulated as

7 $$\frac{\partial c}{\partial t}=\overrightarrow{\nabla }.\left(D \overrightarrow{\nabla }c\right)$$

where $$c=c\left(x,y,z,t\right)$$ is the concentration of the GFP inside the cell. If the diffusion coefficient is set as a function of the spatial coordinate system, Eq. (7) governs any change in the GFP concentration inside the cytoplasm and nucleus. This coefficient depends on both the medium composition and the size of the GFP. Therefore, three values may be associated with the diffusion coefficient: one related to free diffusion inside the nucleus ($${D}_{Nu}=$$ 25 $${\mu m}^{2}/s$$), one to free diffusion inside the cytoplasm ($${D}_{Cyt}=25$$$${\mu m}^{2}/s$$) and one to transport through the NE ($${D}_{NE}^{app})$$^40^. Here, NE is modeled as a permeable membrane whose transport of small molecules through its NPCs is described by simple passive diffusion. To determine the apparent diffusion coefficient of the NE, we considered partition and hindrance factors, $$\varphi$$ and $$\varepsilon$$, respectively. The partition factor is the ratio of the NPC area to the NE area and is calculated by

8 $$\varphi =N\frac{ {\stackrel{\sim }{A}}_{NPC}}{{A}_{NE}}$$

where, ${\stackrel{\sim }{A}}_{NPC}=\left(1/N\right)\sum_{i=1}^{N}{A}_{NP{C}_{i}}$ is the mean transport area of NPCs. The hindrance factor accounts for the resistance of the NPC to the diffusion of molecules, i.e. due to molecular crowding. Therefore, regarding the apparent diffusion coefficient of the NE, we have

9 $${D}_{NE}^{app}=\varphi \varepsilon {D}_{Cyt}$$

Equations (8) and (9) describe how we can model the NE as a virtual permeable membrane. In the simulation of the transport through the NE, we set the value of ${D}_{NE}^{app}$. Our approach toward the problem was to set different values of the diffusion coefficient in the numerical model and find out the values by which we observe a good agreement between the simulation results and FRAP.

Although implicit determination of ${D}_{NE}^{app}$ can be achieved by MD simulation of the transport inside the NPCs, it can also be estimated using FRAP. Considering FRAP, a linear proportionality between the concentration of the molecules and their fluorescence can be assumed^39–41^ by a direct comparison of the results of FRAP and the simulation.

### Governing equations for facilitated transport

Facilitated transport of the GFP-MyoD through the NE is modeled by conserving the mass transport between the nucleus and cytoplasm. To do so, based on Fick’s first law, two diffusion fluxes are considered as

10a $${F}_{x}^{Cyt\to Nu}={{\kappa }_{Cyt\to N} \left[x\right]}_{Cyt}$$

10b $${F}_{x}^{Nu\to Cyt}=-{{\kappa }_{Nu\to Cyt} \left[x\right]}_{Nu}$$

where the positive flux corresponds to the nucleus import of $x$^39^. Thus, the totalflux of $x$ passing through the NE is obtained by summing up the facilitated and passive fluxes. Assuming the same resistance for the passive import and export, we have

11 $${F}_{x}^{tot}={F}_{x}^{Act}+{F}_{x}^{Pass}=\left\{{{\kappa }_{Cyt\to Nu}\left[x\right]}_{Cyt}-{\kappa }_{Nu\to Cyt}{\left[x\right]}_{Nu}\right\}+\left\{\frac{\varphi \varepsilon {D}_{Cyt}{A}_{NE}}{{d}_{NE}}\left({\left[x\right]}_{Cyt}-{\left[x\right]}_{Nu}\right)\right\}$$

which can be rewritten as

12 $${F}_{x}^{tot}=\left({\kappa }_{Cyt\to Nu}+\beta \right){ \left[x\right]}_{Cyt}-\left({\kappa }_{Nu\to Cyt}+\beta \right){\left[x\right]}_{Nu}$$

where, $\beta =\varphi \varepsilon {D}_{Cyt}{A}_{NE}/{d}_{NE}$. The ratio between the two coefficients of the above equation determines the equilibrium state of concentration inside the nucleus and cytoplasm.

### Computational modelling

The numerical simulation of the diffusion mass transfer enables us to study the facilitated and passive transport for two different configurations, Nichoid and Control. We used a commercial program (ANSYS CFX 19) to simulate the mass transfer inside the cells. For both configurations, the geometry consists of two centric ellipsoidal shapes separated by an interface representing the nucleus, cytoplasm and NE, respectively (Fig. 4). These ellipsoids are generated based on the semi axes $a, b\  {\mathrm{and}}\ c$ reported in Fig. 3. Advection terms and time derivatives are discretized by high resolution and second order backward Euler schemes, respectively. This provides an unconditionally stable scheme for the diffusion equation^42^. The convergence criterion for the maximum relative error of discretized equations is set to ${10}^{-6}$. The simulation starts at time zero with the initial condition corresponding to the fluorescence measurement within a time-lapse acquisition after bleaching and continues until an equilibrium state is achieved. We also studied the grid dependency of the apparent diffusion coefficient of NE for both cases and chose a mesh size in a range which enabled us to observe less than 1% variation in results for three coarse, medium and fine elements.

### Simulation of passive transport

For the passive transport, the Newman boundary condition with zero flux is set at the cell membrane. This condition means that, while the GFP can freely move inside the cell by diffusion, the mass inside the cell is conserved and no transport occurs through the cell membrane. This is consistent with the experiment as GFP does not transfer between the cell and the medium, and its fluorescence does not vanish within the FRAP time-frame.

In our numerical approach, NE is modeled as a virtual permeable 20 nm thick membrane with a spatially dependent permeability. Therefore, GFP passes through the NE just by diffusion throughout concentration gradient to reach an equilibrium state. In the case of passive transport, this equilibrium state relates to when the concentration of GFP is uniform all around the cell, which corresponds to a full recovery of the fluorescence after photo-bleaching in the experiment.

### Simulation of facilitated transport

For the facilitated transport, the nucleus and cytoplasm are simulated separately as two different objects but coupled at their interface by Eq. (12). The mass is conserved inside the cell just by imposing zero mass flux at the cell membrane.

As the simulation starts, the initial concentrations of GFP-MyoD inside the nucleus and cytoplasm, and equilibrium ratio, ${R}_{eq}=\left({\kappa }_{Cyt\to Nu}+\beta \right)/\left({\kappa }_{Nu\to Cyt}+\beta \right)$, determine the flux of the GFP-MyoD at each element of the interface (NE). The simulation continues until the total mass flow in all the elements of the interface becomes zero and an equilibrium state is obtained. In this condition, the full recovery of fluorescence after photo-bleaching corresponding to the equilibrium state occurs when ${\left[x\right]}_{Nu}/{ \left[x\right]}_{Cyt}={R}_{eq}$.

## Supplementary Information

Supplementary Information
