# Supplementary material for: The nuclear import of the transcription factor MyoD is reduced in mesenchymal stem cells grown in a 3D micro-engineered niche
Source: Sci Rep. 2021 Feb 4;11:3021. doi: 10.1038/s41598-021-81920-2 (PMC7862644; doi:10.1038/s41598-021-81920-2)
Supplement: Supplementary file 1 — Supplementary Information [file 41598_2021_81920_MOESM1_ESM.docx]

**The nuclear import of the transcription factor MyoD is reduced in mesenchymal stem cells grown in a 3D micro-engineered niche**

**Authors**

Emanuela Jacchetti ^1§*^, Ramin Nasehi^1§^, Lucia Boeri^1^, Valentina Parodi^1^, Alessandro Negro^2^, Diego Albani^3^, Roberto Osellame^4,5^, Giulio Cerullo^5^, Jose F Rodriguez Matas^1^, Manuela Teresa Raimondi^1^.

*
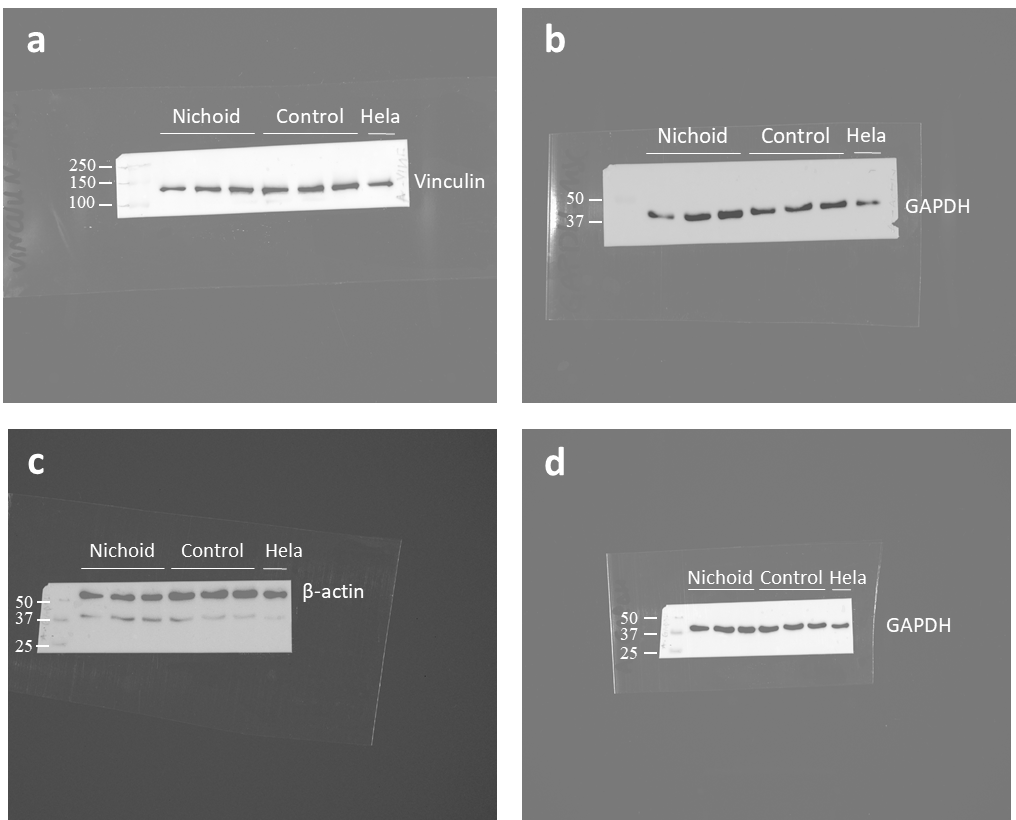
*

**Supp. 1| Full length western blots.**

*These images are the original, unprocessed merged versions of the chemiluminescent signals in blots of figure 1.e and 2.e with the relative colorimetric acquisitions. These versions show the whole full length blots with the relative chemiluminescent signals and protein standard bands.* ***a)*** *Blot image corresponding to the vinculin expression level in MSCs grown into the Nichoid and on flat substrate.* ***b)*** *Blot image corresponding to the GAPDH expression level in MSCs grown into the Nichoid and on flat substrate. These data are the internal loading control for the normalization of vinculin expression levels (a).* ***c)*** *Blot image corresponding to the β-actin expression level in MSCs grown into the Nichoid and on flat substrate. The lower bands are the residual bands of the previous GAPDH detection (d).* ***d)*** *Blot image corresponding to the GAPDH expression level in MSCs grown into the Nichoid and on flat substrate. These data are the internal loading control for the normalization of actin expression levels (c). The 7th bands of all the blots correspond to Hela cells grown on standard culture dishes. These data were not used for this paper since they were part of another experiment. The chemiluminescent signals and the relative colorimetric and merged images were acquired using the ChemiDoc Imaging System (Biorad). The signal intensity was analysed using ImageJ software.*

***
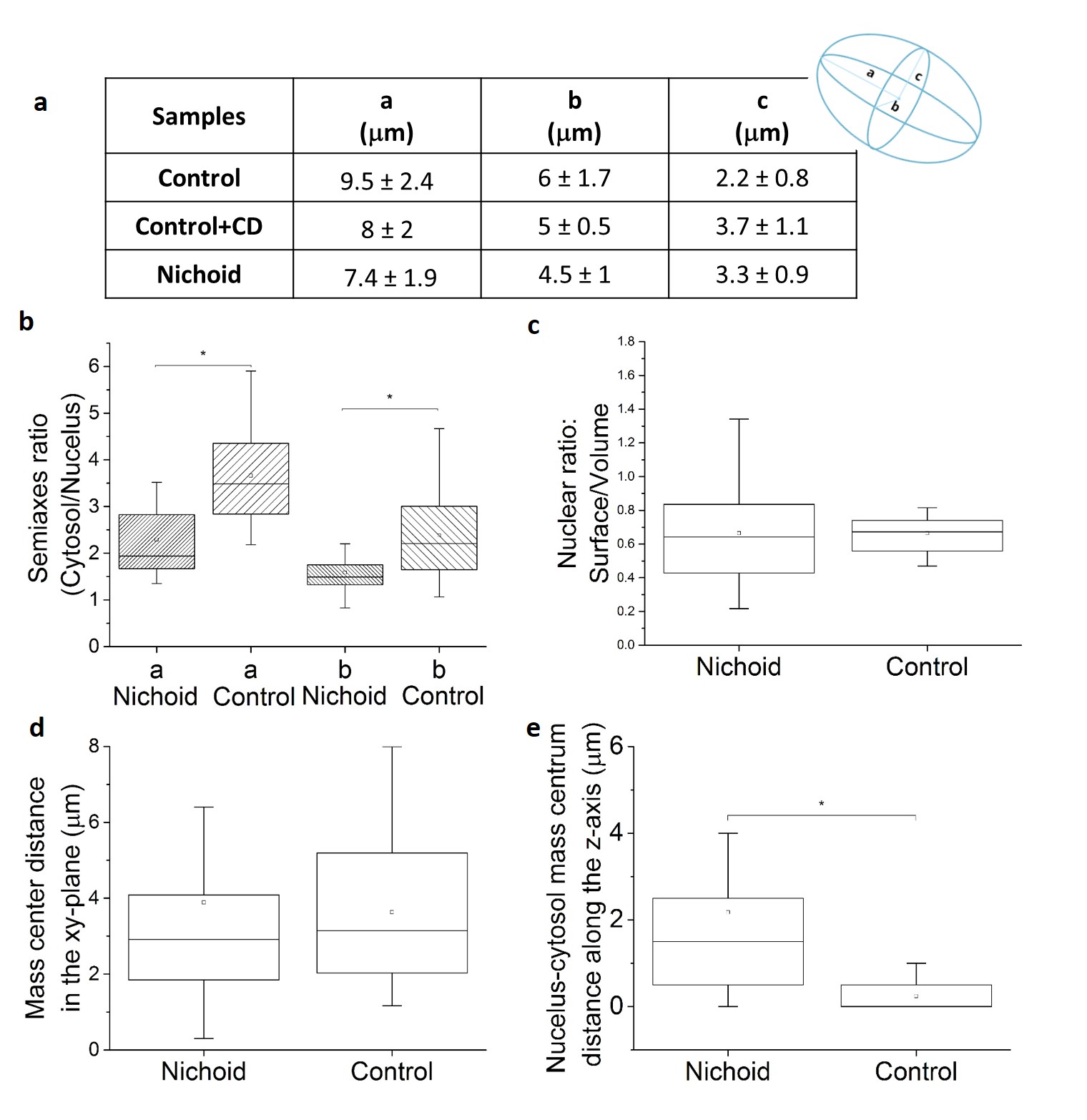
***

**Supp. 2| Spatial organization of cell morphology inside the Nichoid artificial niche.**

**a** *Table summarizing the results obtained on the nuclear semiaxes lengths along the three directions (xyz) for MSCs grown on the Control with and without the Cytochalasin-D treatment and on MSCs grown into the Nichoid. Data shows that the inhibition of actin polymerization modifies the cell nuclear shape from a thin disk to a prolate ellipsoid configuration.* **b** *Semi-axes (cellular/nuclear) ratio. The graph highlights the portion of cytoplasm around the cell nucleus in the xy plane, that is bigger in spread cells respect to the cells grown into the Nichoid.* **c** the *ratio between nuclear surface and its volume do not significantly change with cell morphology*. **d** *Mass centrum distance calculated in the cell equatorial plane (xy): this distance is not significantly affected by the two substrates.* **e** *Mass centrum distance calculated in the cell longitudinal plane (xz): it is only appreciable in MSCs grown into the three-dimensional scaffold. On the flat substrate the two centers of mass coincide summarizing the results obtained on the nuclear semi axes lengths along the three-xyz directions for MSCs grown into the Nichoid, on the Control and on cells grown on the Control but treated with cytochalasin D. The inhibition of actin polymerization modifies the cell nuclear shape to a prolate ellipsoid configuration.*


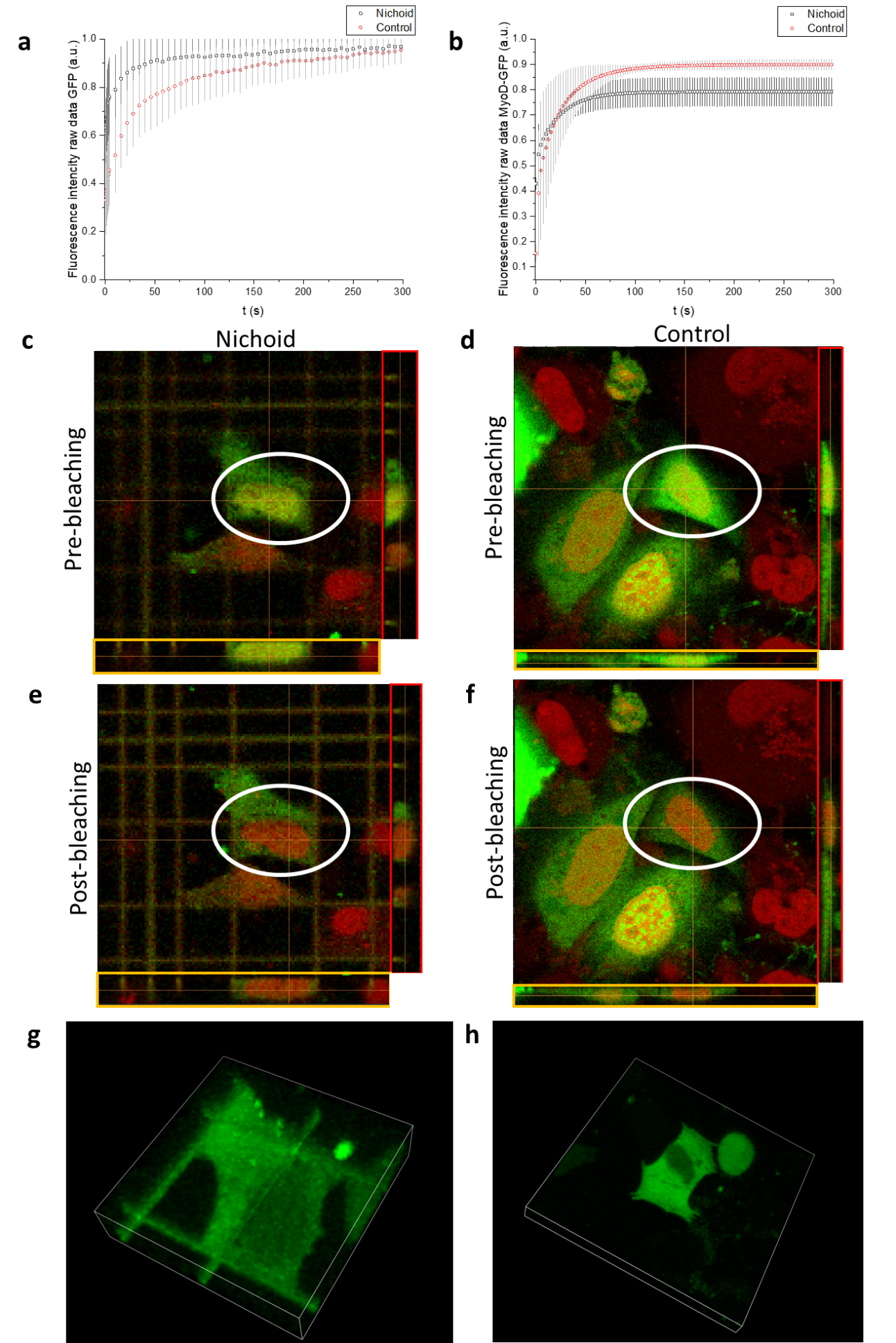


**Supp. 3| FRAP experiments data and visualization**

**a, b** *Nuclear fluorescence recovery after photobleaching of GPF protens (****a****) and MyoD-GFP proteins (****b****) of MSC grown in the Nichoid (black) and on the Control (red).* *Data are reported as average and SEM. Graph reports a downsampling with respect to the imaging acquisition.* ***c, d*** *Planar section plus orthogonal (yellow ROIs) and sagittal (red ROIs) projection of pre bleached GFP-expressing MSC grown into the Nichoid (****c****) and on Control (***d***). In green it is visible the GFP protein distribution and in red it is identified the cell nucleus (Draq5 dye). The co-localization of GFP and red dye results yellow nuclei. The circular ROI highlights the cell under investigation.* ***e, f*** *Planar section plus orthogonal (yellow ROIs) and sagittal (red ROIs) projection of GFP-expressing MSC grown into the Nichoid (****e****) and on Control (***f***) after photo-bleaching. In this case the cell cytoplasm is green and the cell nucleus red because the GFP inside the cell nucleus is bleached.* ***g, h*** *3D reconstruction of GFP-expressing MSC grown into the Nichoid (****g****) and on the Control (****h****) around 200 s after photo-bleaching .*
